# Predicting Peritoneal Dissemination of Gastric Cancer in the Era of Precision Medicine: Molecular Characterization and Biomarkers

**DOI:** 10.3390/cancers12082236

**Published:** 2020-08-10

**Authors:** Yanyan Chen, Quan Zhou, Haiyong Wang, Wei Zhuo, Yongfeng Ding, Jun Lu, Guanghao Wu, Nong Xu, Lisong Teng

**Affiliations:** 1 Department of Surgical Oncology, The First Affiliated Hospital, School of Medicine, Zhejiang University, Hangzhou 310003, China; 3100104153@zju.edu.cn (Y.C.); zhouquanzq@zju.edu.cn (Q.Z.); lanceter1@zju.edu.cn (H.W.); 11218203@zju.edu.cn (J.L.); 2 Department of Cell Biology, School of Medicine, Zhejiang University, Hangzhou 310058, China; 0012049@zju.edu.cn; 3 Department of Medical Oncology, The First Affiliated Hospital, School of Medicine, Zhejiang University, Hangzhou 310003, China; dingyongfeng@zju.edu.cn (Y.D.); nongxu@zju.edu.cn (N.X.); 4 School of Medicine, Hangzhou Normal University, Hangzhou 311121, China; ywwgh0945@sina.com

**Keywords:** gastric cancer, peritoneal dissemination, diffuse type gastric cancer, molecular classification, biomarker

## Abstract

Gastric cancer (GC) is a leading cause of worldwide cancer-related death. Being a highly heterogeneous disease, the current treatment of GC has been suboptimal due to the lack of subtype-dependent therapies. Peritoneal dissemination (PD) is a common pattern of GC metastasis associated with poor prognosis. Therefore, it is urgently necessary to identify patients at high risk of PD. PD is found to be associated with Lauren diffuse type GC. Molecular profiling of GC, especially diffuse type GC, has been utilized to identify molecular alterations and has given rise to various molecular classifications, shedding light on the underlying mechanism of PD and enabling identification of patients at higher PD risk. In addition, a series of diagnositc and prognostic biomarkers of PD from serum, peritoneal lavages and primary GCs have been reported. This comprehensive review summarizes findings on the multi-omic characteristics of diffuse type GC, the clinical significance of updating molecular classifications of GC in association with PD risk and research advances in PD-associated biomarkers.

## 1. Introduction

Gastric cancer (GC) is the world’s fifth most commonly diagnosed cancer and third leading cause of cancer-related mortality [1]. The vast majority of GCs are adenocarcinomas, which are traditionally divided into intestinal and diffuse types based on Lauren classification [2]. However, GC is both genotypically and phenotypically highly heterogeneous, and the clinical utility of histological classification is limited. Due to the lack of individualized targets, treatment for GC is currently selected mainly based on disease stage, resulting in unsatisfactory outcomes. Next-generation sequencing (NGS) enables the high-throughput and systematic analysis of genetic alterations, offering novel insights into cancer mechanisms. Evolving sequencing techniques provided molecular perspectives into the genotypical heterogeneity of GC, uncovered multiple driver aberrations and gave rise to molecular classifications. For example, The Cancer Genome Atlas (TCGA) categorized GC into four subtypes: Epstein–Barr virus positive (EBV), microsatellite instable (MSI), genomically stable (GS) and chromosomal instability (CIN) [3]. Subsequently, the Asian Cancer Research Group (ACRG) described four subtypes of GC, MSI, MSS/EMT (microsatellite stable/epithelial-to-mesenchymal transition), MSS/TP53+ (TP53 active) and MSS/TP53− (TP53 inactive), based on gene expression profiles [4]. These molecular classifications identified GC subtypes with distinct genotypes and phenotypes, which, in turn, drives research into potential therapeutic targets and subtype-directed management [5].

Peritoneal dissemination (PD) is a frequent pattern of metastasis in advanced GC. PD can occur in different clinical scenarios. Synchronous PD is found at the time of initial GC diagnosis or occasionally during intended radical surgery, while metachronous PD occurs as peritoneal recurrence after radical surgery, accounting for up to 50% of all recurrences [6]. In either situation, occult PD presents concurrently; it is often invisible and easily missed on clinical imaging scans. Peritoneal lavage cytology in staging laparoscopy can help detect disseminated cancer cells in the peritoneal cavity before formation of gross peritoneal carcinomatosis, which has allowed the definition of PD to be updated as cytology positive (CY1) [7]. However, the accuracy of peritoneal lavage cytology is limited [8]. A recent study incorporated computed tomography (CT) phenotypes and the Lauren subtype to detect occult PD [9]. Through the past decade, emerging studies have proposed novel biomarkers for detecting synchronous PD and predicting metachronous PD in addition to peritoneal cytology and clinical imaging [10].

Patients with PD often develop complications such as bowel obstruction and malignant ascites and have unsatisfactory response to chemotherapy and poor overall survival (reported median overall survival 5–16 months) [11,12,13,14]. For GC patients at high risk of PD, the evolving intraperitoneal chemotherapy, including adjuvant hyperthermic intraperitoneal chemotherapy (HIPEC) and early postoperative intraperitoneal chemotherapy (EPIC), have provided an encouraging trend towards improved disease outcomes [15]. Therefore, risk prediction of PD has been essential in GC management. A previous study revealed that risk factors for metachronous peritoneal carcinomatosis of GC included serosa invasion (T3-4), nodal metastasis, signet ring cell and undifferentiated gradings [16]. Similarly, female gender, advanced T-stage, diffuse type, type IV of the morphology-based Borrmann’s classification, and venous invasion have been found to be risk factors for peritoneal recurrence after radical resection [14,17,18,19]. The observed association between PD and female gender might be due to the high frequency of diffuse type GC or signet ring cell carcinoma (SRCC) in female patients [20]. Notably, Borrmann type IV and Lauren diffuse type GC are associated with higher risk of PD and concurrent lower risk of liver metastasis; meanwhile, intestinal type GC shows an opposite metastatic pattern, implying distinct underlying mechanisms in regard to these disease subtypes [21,22]. However, due to the biological heterogeneity of GC observed even within one histological type, the known clinical and pathological characteristics may not fully represent the potential of PD. For example, peritoneal recurrence also develops in a considerable subgroup of intestinal type GC [21]. From a molecular biological perspective, the PD process is associated with epithelial-mesenchymal transition (EMT) and gain of invasive and angiogenetic properties of cancer cells in the context of peritoneal components. The progress in mechanistic studies of PD is reviewed in detail elsewhere [23,24,25]. In the era of precision medicine when novel NGS-based molecular classifications of GC have been recognized, it is of urgent priority to identify molecular characteristics associated with PD risk in GC patients after or even before curative resection, which might improve treatment decision and disease outcomes.

The present review summarizes evidence on clinical significance of multi-omic characteristics of diffuse type GC and the updated molecular classifications of GC. Specifically, this review considers disease subtypes associated with PD risk across different classification systems which differentiate an aggressive subset of GC carrying high risk of PD. Finally, advances in research on diagnostic or predictive PD biomarkers are summarized. 

## 2. Diffuse Type Gastric Cancer from a Molecular Perspective

**Figure 1:**
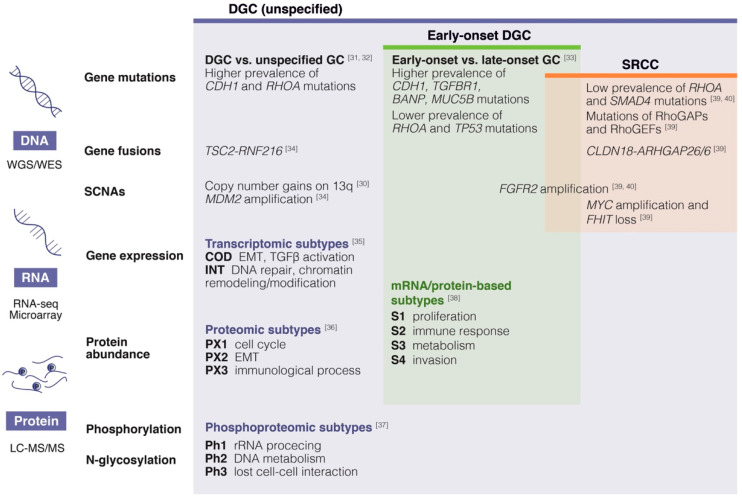
Genomic, transcriptomic and proteomic alterations of diffuse type gastric cancer [30,31,32,33,34,35,36,37,38,39,40]. DGC, diffuse type gastric cancer; EMT, epithelial-to-mesenchymal transition; LC-MS/MS, liquid chromatography-tandem mass spectrometry; SCNA, somatic copy number alteration; SRCC, signet-ring cell carcinoma; WGS/WES, whole genome sequencing/whole exome sequencing. Reference numbers are shown in square brackets.

Lauren classification distinguishes three major histological subtypes of GC: intestinal, diffuse and mixed. Diffuse type GC (DGC), which accounts for approximately 30% of all GC cases, corresponds to the “poorly cohesive carcinoma” category defined by the World Health Organization [26]. Whether diffuse type GC (including SRCC) is associated with poorer outcomes compared to other subtypes remains subject to debate [27]. However, it is well-established that diffuse type GC is associated with higher risk of PD compared to the intestinal type [28,29]. Notably, a recent study revealed that Lauren histologic type was the most significant risk factor for postoperative recurrence. Furthermore, diffuse type was associated with peritoneal recurrence while the intestinal type was associated with distant recurrence [21]. These findings suggest that further investigations into DGC may help elucidate biological mechanisms of PD. Novel techniques, including genomic, transcriptomic and proteomic profiling, provided novel insights into cancer biology and intrinsic heterogeneity of this histological subtype (Figure 1).

### 2.1. Multi-Omic Characterization of Diffuse Type Gastric Cancer

In GC, the level of loss of heterozygosity (LOH) is associated with histologic type. High-level LOH correlates with intestinal or mixed-type GC, whereas a baseline-level LOH, involving zero to one chromosome, was frequent with diffuse type GC, suggesting less extent of genome instability in DGC [41,42]. In particular, copy number gains on 13q have been reported to associate with diffuse GC [30].

Presence of germline or somatic *CDH1* mutation is considered a hallmark of DGC. Inactivation of *CDH1* leads to hereditary diffuse GC and modulates E-cadherin, Wnt or RHOA signaling, affecting cell invasion and migration in GC [43,44]. Somatic *CDH1* mutation has been associated with reduced patient survival in DGC [45]. Whole genome sequencing (WGS) studies have revealed recurrent somatic alterations in *TP53*, *CDH1* and *RHOA* in DGC [31]. In another study, 33% (29/87) of DGC and only 2% (1/50) of intestinal GC cases had *CDH1* mutation. *RHOA* gain-of-function mutation was observed in 25% (22/87) of DGC cases (compared to none among 51 intestinal GC cases) [32]. However, there is no evidence to suggest that *RHOA* mutation affects survival [33,46]. In addition, *MDM2* gene amplification and *TSC2-RNF216* fusion were reported in DGC [34]. A WGS-based study including 49 GC cases showed that DGC is characterized by a significantly lower clonality and smaller number of somatic and structural variants compared with the intestinal subtype, suggesting genome stability of DGC [47].

A recent study by Kim et al. identified a molecular signature through transcriptomic profiling which discriminated two subtypes of DGC: the intestinal-like (INT) and core diffuse-type (COD) subtypes. In this study, EMT-related expression signature was the key factor of subtype dichotomization. Combined survival analysis of three cohorts revealed patients with the COD subtype had lower overall survival and more significant response to adjuvant chemotherapy. In contrast, the INT subtype, associated with a higher tumor mutation burden (TMB), enrichment in upregulated CD274 and aberrant DNA damage repair pathway, was predicted to respond better to immune checkpoint inhibitors [35].

Ge et al. divided DGC into three subtypes (PX1-3) based on proteomic signature data from 84 patients. Subtypes enriched in cell cycle (PX1), EMT (PX2) and immunological process (PX3) were distinctly associated with clinical outcomes. Patients with PX3 had the shortest overall survival alongside poor response to chemotherapy [36]. In a subsequent study, the same research group mapped the phosphoproteomic landscape of 83 DGC cases, enabling a novel molecular subtyping (Ph1 to Ph3) that correlated with multiple clinical features, overall survival and chemosensitivity. Comparison of PX1-3 with Ph1-3 revealed agreement of two classification systems [37]. Even though various mechanisms related to metastasis, such as EMT, cell-cell interaction and GTPase signaling [24,48], were observed in different proteomics-based subtypes, neither study addressed the risk of PD or other distant metastasis patterns in these subtypes.

### 2.2. Early-Onset Diffuse Type Gastric Cancer

GC diagnosed in young patients (under the age of 40 or 45 years) is referred to as “early-onset” disease, which has been reported to strongly correlate with female gender, diffuse histology, Borrmann type IV and higher prevalence of peritoneal metastasis (9.81% vs. 4.98% in early-onset vs. other patients, respectively) [49,50]. Molecular features of early-onset DGC have been described in NGS studies. A WES-based study found a higher prevalence of somatic *CDH1* (42.2% vs. 17.4%) and *TGFBR1* (7.3% vs. 0.9%) mutations and lower prevalence of *RHOA* (9.2% vs. 19.1%) mutations in sporadic early-onset than in late-onset DGC cases. Notably, *CDH1* mutation was associated with shorter survival [33]. A multi-omic study of early-onset GC in a cohort that comprised mostly DGC cases (74/80) defined four subtypes that were associated with mRNA/protein signatures of proliferation, immune response, metabolism and invasion, respectively [38].

### 2.3. Gastric Signet Ring Cell Carcinoma

As a subset of DGC with unique morphology, gastric SRCC has been associated with younger age of onset and female predominance compared with other histological subtypes [51,52]. Although conferring favorable prognosis at an early stage, SRCC has worse prognostic impact as disease progresses to advanced stages [27,39]. Importantly, SRCC is associated with higher rate of both synchronous and metachronous PD [53]. In a phase 2 clinical trial of pembrolizumab, none of 10 patients with metastatic gastric SRCC responded to this treatment [54]. Yang et al. investigated the molecular characteristics of 32 SRCC cases in a WGS study, reporting high frequency of *CLDN18-ARHGAP26/6* fusion that was concurrently associated with relatively poorer outcomes and resistance to oxaliplatin/fluoropyrimidines-based chemotherapy. In vitro assays illustrated that *CLDN18-ARHGAP26/6* fusion could induce chemotherapy resistance in GC cells. The group also reported that some mutations enriched in DGC (*RHOA* and *SMAD4*) were infrequent in SRCC [39]. Likewise, Kwon et al. reported the infrequency of *RHOA* and *SMAD4* mutations in SRCC compared to other types of poorly cohesive carcinomas [40].

### 2.4. Molecular Features of Diffuse Type Gastric Cancer and Peritoneal Dissemination

Multi-omic profiling and the recently-purposed classifications of DGC revealed heterogeneity within this disease. Genetic alterations of *CDH1* and loss of E-cadherin was a remarkable event in DGC. Previous studies revealed that somatic CDH1 mutation and reduced E-cadherin expression were predictive of poor survival [55,56]. Correspondingly, EMT-related signature, in which reduced E-cadherin expression is a key component, has been found in some subsets of DGC in transcriptomic- or proteomic-based classification. As EMT is a critical biological process for PD, it is likely that these subsets of DGC carry higher risk of PD. Moreover, particular subsets of DGC might present features similar to those of intestinal type GC, thus, they are likely to be at relatively lower risk of PD. Nevertheless, association between molecular features of DGC and PD risk requires further validation.

## 3. Molecular Classifications of Gastric Cancer and Subsets with Peritoneal Dissemination Propensity

Application of NGS techniques enables establishment of various molecular classification systems for GC [5,57]. Landmark genomic and expression-based classifications were proposed by The Cancer Genome Atlas (TCGA) and Asian Cancer Research Group (ACRG), respectively. In recent years, expressional and proteomic signatures based on the tumor microenvironment (TME), including stromal and immune composition, have allowed novel classifications of GC. This section discusses the biological and clinical implications of current molecular classifications of GC, especially focusing on the subsets associated with PD.

### 3.1. Expression Microarray-Based Classifications

Exploratory analyses based on expression profiles tested using a microarray were conducted to classify GC subtypes with distinct molecular signatures that also show prognostic significance [58,59,60]. Other studies have investigated the association between expression-based classifications and PD propensity. Motoori et al. constructed a prediction system for peritoneal metastasis using 18 informative genes identified using an expression profile of 30 primary GC cases, with prediction accuracy of 75% in a validation cohort of 24 patients [61]. Takeno et al. established a 22-gene expression profile that was associated with peritoneal relapse after curative surgery in 56 GC patients, which yielded an overall accuracy of 76.9% in relapse prediction (*N* = 85) [62]. However, there was no overlap between the two discussed gene sets, which might be partly explained by the different inclusion criteria for the exploration cohort between the studies.

The Singapore-Duke group proposed two intrinsic genomic subtypes with distinct patterns of gene expression: genomic intestinal (G-INT) and genomic diffuse (G-DIF). The intrinsic subtypes showed a concordance of 64% with Lauren histopathological subtypes. G-DIF tumors were associated with poorer survival compared with G-INT tumors in patients following adjuvant 5-fluorouracil-based therapy [63]. In a follow-up study, the same group proposed a new model that divides GC into proliferative, metabolic and mesenchymal subtypes. The mesenchymal subtype was associated with the Lauren diffuse subtype, characterized by high activity of the EMT and angiogenesis pathway. The study further suggested that the mesenchymal subtype was likely associated with sensitivity to PI3K-AKT-mTOR inhibitors [64]. Given the concordance between Singapore-Duke proposals and Lauren classification, it is likely that G-DIF and mesenchymal subtypes were associated with higher PD risk, though further research is required to validate this connection.

### 3.2. The Cancer Genome Atlas (TCGA) and Other Genome-Based Classifications

In 2014, TCGA research network published comprehensive identification of genetic alterations associated with GC, integrating data from six different platforms of molecular testing in 295 primary gastric adenocarcinomas. Four GC subtypes, EBV, MSI, GS and CIN, were determined. Specifically, the GS subtype was enriched for Lauren diffuse type, *RHOA* mutations or fusions involving Rho-family GTPase-activating proteins; *CDH1* and *ARID1A* somatic mutations were also common in the GS subtype. In addition, the GS subtype exhibited markedly elevated expression of cell adhesion and angiogenesis-related pathways but reduced activity of mitotic pathways such as p53 signaling and Aurora A/B signaling compared with other subtypes [3]. TCGA classification was a landmark of GC molecular classification and its clinical significance has been demonstrated. For instance, Sohn et al. investigated patient prognosis in regard to TCGA subtypes in two independent cohorts *(N* = 267 and 432), revealing that among all described types, the GS subtype was associated with the worst prognosis and the patients with GS tumor benefit the least from adjuvant chemotherapy [65]. A phase 2 clinical trial of pembrolizumab in metastatic GC reported dramatic response in all patients with MSI (*N* = 6) and EBV (*N* = 4) subtypes, but the response rate was low in GS (12%, *N* = 25) and CIN (5%, *N* = 20) [54]. In 2018, an update of TCGA classification was reported in the context of gastrointestinal adenocarcinomas, whereby a hypermutated single-nucleotide variant (HM-SNV) subtype, the majority of which harbors *POLE* mutation, was added to the previous system [66]. HM-SNV tumors are microsatellite and genome stable and associated with early-onset disease, suggesting they may exhibit clinical features similar to those of the GS subtype.

Apart from TCGA, other genome-based classifications of GC have been proposed. Chen et al. performed WES of 78 Chinese GC cases, dichotomizing GC into high-clonality (HiC) and low-clonality (LoC) subsets based on the number of intratumoral subclones [67]. Li et al. recognized two subtypes with distinct mutational signatures by analyzing mutational data from 544 GC patients [45]. Both classifications showed prognostic significance within the study cohorts, but their broader clinical implications require further validation.

### 3.3. Asian Cancer Research Group (ACRG) Classification

In 2015, ACRG categorized GC into four subtypes based on gene expression data from 300 cases, namely MSI, MSS/EMT, MSS/TP53+ and MSS/TP53−. MSS/EMT, the mesenchymal-like subtype, was associated with the worst prognosis and characterized by a gene expression signature related to EMT. Moreover, this subtype was characterized with early onset, predominance of Lauren diffuse type (37/46), a high proportion of SRCC cases (20/46), a more advanced AJCC stage and a higher incidence of peritoneal seeding as the first recurrence site compared to other subtypes (64.1% vs. 22.7%) [4]. Notably, despite a series of shared characteristics, *CDH1* and *RHOA* mutations were infrequent in the MSS/EMT subtype, suggesting this subtype was not equivalent to the TCGA GS subtype. Detailed comparison of TCGA and ACRG classifications was discussed in the original ACRG publication and reviewed by Serra et al. [68]. Briefly, ACRG classification has significant relevance with clinical outcomes and was the first to inform a link between molecular subtype and distinct recurrence patterns after surgery. In addition to a tendency to develop PD, ACRG also reported that patients of the MSS/EMT subtype rarely develop liver metastases as the first recurrence site, and this pattern was also observed in DGC [21] and Borrmann IV GC [22]. The tendency of peritoneal recurrence in the MSS/EMT subtype is consistent with the established role of EMT in the peritoneal spread of cancer cells. In terms of response to immunotherapy, a clinical trial revealed that the MSS/EMT subtype responded poorly to pembrolizumab (0% vs. 30.7% in MSS/EMT subtype vs. non-MSS/EMT subtype, respectively) [54].

Attempting to reproduce molecular classifications, Setia et al. examined 14 protein and mRNA expression biomarkers and identified five subgroups of GC characterized by Epstein–Barr virus positivity, microsatellite instability, aberrant E-cadherin and p53 expression corresponding to molecular subtypes in TCGA and ACRG systems (*N* = 146). GC with aberrant E-cadherin corresponding to *CDH1*-mutated subtype was predominantly the diffuse type. However, this subgroup presented no significant survival difference compared to other GC types in the study cohort [69]. Ahn et al. replicated this study using IHC/ISH of a tissue microarray in a large Asian cohort (*N* = 349), revealing significantly poorer overall survival in the aberrant E-cadherin subtype than in the other four subtypes [70]. Another study by Pinto et al. validated this method and integrated it with NGS in a Chilean cohort (*N* = 91) and proposed an improved low-cost stratification system for GC patients that includes assessment of HER2 and PDL1 status [71]. This subtyping system provided a simplified and cost-effective method to reproduce the known molecular classifications. However, the limited biomarkers included in this method were inadequate to fully represent the molecular features of GC. For example, in relation to PD, only aberrant E-cadherin has been used as the biomarker of the subtype corresponding to ACRG-MSS/EMT subtype. Further validation and refinement of this method based on NGS are required.

### 3.4. Tumor Microenvironment-Based Signatures

Over the last two decades, there has been a fundamental paradigm shift in the understanding of the local TME. A tumor is considered as a reconstructed “tissue” or even “organ” with a full set of blood supply, stromal support and immune cell infiltration [72]. Therefore, analysis of TME-based molecular signatures is highly informative of cancer progression mechanisms, treatment response and clinical outcomes.

In GC, high intra-tumoral stroma proportion, more commonly seen in diffuse type GC, has been indicated to be predictive of unfavorable prognosis [73]. In 2016, a stromal-based GC classification was established, in which four phenotypes were recognized, representing vascular and immune status. These phenotypes were vascular immature/noninflammatory (VINI), inflammatory (I), vascular mature/inflammatory (VM/I) and vascular mature (VM). These phenotypes exhibit differentiated enrichment of immune response signatures. Patients with vascular mature GC (VM or VMI phenotype) had poorer overall survival than their counterparts with other phenotypes. Although neither metastasis rate nor pattern was directly compared between subgroups, comparison with ACRG classification revealed that GC of the MSS/EMT subtype presented either the VM or VMI phenotype, implying a linkage between angiogenic signature (vascular mature) and PD propensity [74].

The advent of cancer immunotherapy represented by the immune checkpoint inhibitors (ICIs) also involved treatment of GC [75], thus, immune TME of GC has drawn increasing interest of oncologists. Immunity signatures in GC were found to be significantly associated with overall survival and treatment response [76]. An immune response expression signature consisting of 29 genes divided GC into subgroups associated with status of PD-L1, EBV, MSI and tumor infiltrating lymphocytes status, which showed prognostic significance [77]. Pan-cancer analysis of the TME in TCGA samples characterized six immune subtypes that impact prognosis, among which, C1 (wound healing) and C2 (IFN-gamma dominant) subtypes comprised the majority of TCGA GC cases [78]. Despite associations with survival and therapeutic response, none of these immunity-based subtypes have been reported to have clinicopathological features corresponding to propensity of peritoneal metastasis.

### 3.5. Molecular Profiling of Peritoneal Metastases or Malignant Ascites

So far, the majority of molecular classifications of GC have been developed based on profiling of primary tumors. However, Pectasides et al. reported a significant discordance of targetable alterations between primary and metastatic gastroesophageal adenocarcinomas [79], thus, molecular profiling of peritoneal metastases or malignant ascites will complement the understanding of PD. Hu et al. investigated malignant ascites-derived exosomes from GC patients with PD and identified a signature containing 29 exosomal miRNAs that might be informative of PD mechanisms [80].

Wang et al. first described the genomic and immune landscape of peritoneal metastases of GC in a multi-omic study that included 43 cases. Integrative analysis of WES, RNA-seq and the immune profile revealed two molecular subtypes, ‘mesenchymal-like’ (M) and ‘epithelial-like’ (E) with discriminating response rates to chemotherapy (31% vs. 71% in M vs. E subtype, respectively). No significant difference in survival was observed between the subtypes as all patients in the cohort had short survival. Compared to the E subtype, the M subtype had higher expression of mesenchymal signature genes, lower expression of epithelial markers, and it was more genomically and chromosomally stable. Moreover, this subtype had higher expression of TGF-β pathway genes, suggesting an immune suppressive microenvironment. Immune checkpoint TIM-3, its ligand galectin-9 and VISTA were highly expressed in the M subtype, which could be potential therapeutic targets [81]. To elucidate the connection between the molecular features of peritoneal metastases and the known molecular classifications of primary GC and, in the meanwhile, to further explore the mechanisms of PD, large scale NGS-based studies comparing primary lesions and corresponding peritoneal metastases remain required.

### 3.6. Subsets Associated with Peritoneal Dissemination Propensity Across Multiple Classifications

**Figure 2:**
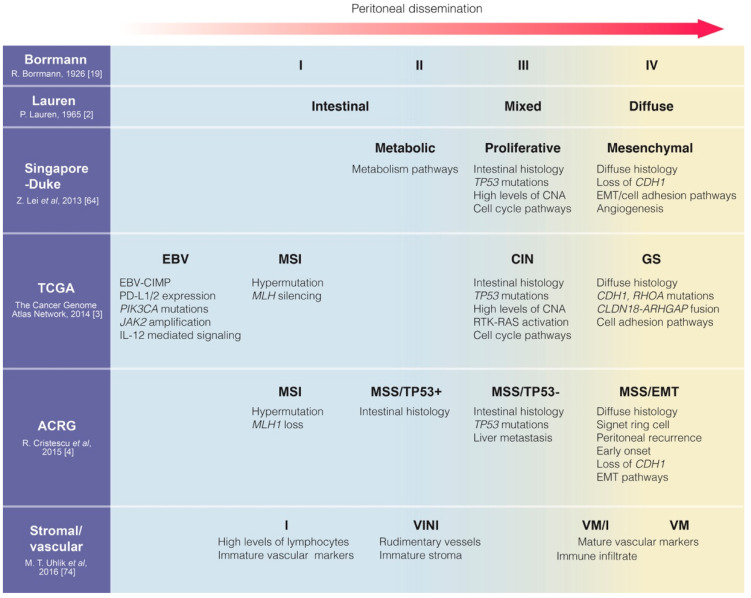
Molecular classifications of gastric cancer in association with propensity of peritoneal dissemination [2,3,4,19,64,74]. ACRG, Asian Cancer Research Group; CIN, chromosomal instability; CNA, copy number alteration; EBV, Epstein–Barr virus positive; EMT, epithelial-to-mesenchymal transition; GS, genomically stable; I, inflammatory; MSI/MSS, microsatellite instable/microsatellite stable; TCGA, The Cancer Genome Atlas; VINI, vascular immature/noninflammatory; VM, vascular mature; VMI, vascular mature/inflammatory. Reference numbers are shown in square brackets.

There is remarkable similarity between some molecular subtypes across different classification systems, such as the mesenchymal subtype of Singapore-Duke, the GS subtype of TCGA and MSS/EMT of ACRG (Figure 2). These subtypes can be characterized by clinicopathological features including early onset, female-predominance, diffuse histology, SRCC/undifferentiated morphology, relatively poor survival and response to chemotherapy and, most importantly for this review, the propensity of PD. From the molecular perspective, each of these GC subtypes may harbor one or more of the following features: genomic inactivation of *CDH1*/loss of E-cadherin, *RHOA* mutations, *CLDN18-ARHGAP* fusion, genome stability, EMT- and angiogenesis-related expression signatures. Although not equivalent, these subtypes partially overlap, suggesting the existence of a typical GC subset associated with high risk of PD. The common molecular features in this subset may also provide additional knowledge of mechanisms related to PD propensity in GC. While not all subtyping systems exhibited prognostic significance, identification of this particular subset of GC patients at high risk of PD may instruct clinical decisions, including use of adjuvant intraperitoneal chemotherapy and recommendation for careful follow up.

Notably, the GC subtypes associated with PD propensity are mutually exclusive with EBV-associated or MSI subtypes, which are the known markers of ICI response. Galon et al. suggested a novel four-category classification of tumors based on T cell infiltration, hot, altered-excluded, altered-immunosuppressed and cold, among which the “cold tumors” were characterized by absence of T cell infiltration, low TMB, poor antigen presentation and insensitivity to T cell killing [82]. Based on these molecular features, we postulate that GCs with high PD propensity are predominantly “immune cold tumors”. Apart from intraperitoneal chemotherapy, candidate treatment of “immune cold tumors” includes CAR-T cell therapy, vaccine-based therapy and oncolytic therapy, which may sensitize GCs to ICIs.

## 4. Molecular Markers for Detection or Prediction of Peritoneal Dissemination

Early detection of peritoneal micrometastasis for GC mainly relies on staging laparoscopy with peritoneal cytology, which offers prognostic significance [83,84]. In approximately 10% of patients without gross metastases, CY1 was present as the only evidence for advanced disease [85,86]. Patients with CY1 can be treated using conversion therapy (defined as surgical resection aimed at achieving R0 after chemotherapy for advanced tumors that were originally either unresectable or marginally resectable), which leads to prolonged survival [87]. Nevertheless, its diagnosis accuracy of early PD is still limited, with reported sensitivity of 64% to 89% [7,8]. Furthermore, a previous study has shown that up to 25% of patients with baseline negative cytology develop PD following radical resection and systemic chemotherapy [14]. As a result, much research effort has been dedicated to searching for molecular markers to detect synchronous (occult) PD and/or predict metachronous (recurrent) PD. Such candidate biomarkers include epithelial markers, tumor-associated antigens, proteins involved in angiogenesis, EMT, cell survival and metabolism (Table 1).

### 4.1. Serum Tumor Markers

Serum tumor markers, particularly CEA, CA19-9 and CA72-4, are established biomarkers for diagnosis and disease-progress monitoring in GC patients [88]. Accumulating evidence has shown significant association between elevated CEA [89], CA19-9 [89,90,99,102], CA72-4 [88,99,102] and CA12-5 [90,99,100,101] levels and synchronous and/or metachronous peritoneal metastasis. Specifically, CA12-5 level has shown a higher predictive performance for PD and a correlation with the degree of PD as well as the presence of malignant ascites [90,100]. Apart from traditional serum tumor markers, CCL5 level has been reported as a potential biomarker for occult peritoneal metastasis [103].

### 4.2. Molecular Markers in Peritoneal Lavages

Assessment of biomarkers in peritoneal lavages, which is obtained in standard staging laparoscopy, can complement findings from peritoneal cytology. Reverse-transcriptase polymerase chain reaction (RT-PCR) detection of a series of biomarkers in peritoneal lavages could increase the sensitivity of PD detection (38–100% vs. 12.3–67% respectively, as reviewed by Virgilio et al. [134]). Among them, CEA was the most frequently used [91,92,93,94,95,96,97,98]. Other biomarkers include CA12-5 [98], CK20 [104], Ber-EP4 [116,117,118,119], MMP-7 [123,124], survivin [91], MUC2 [91,126], IL-17 [121], FABP1, TFF1 and MASPIN [126]. Most diagnostic biomarkers for PD were also prognostic. For patients with negative cytology, positivity of multiple mRNA markers could predict peritoneal recurrence and an unfavorable outcome. Cohort studies showed that positive CEA alone [94,95], and in combination with CK20, predicts peritoneal recurrence and shorter survival [104,105,106,107,108]. A Japanese study revealed that preoperative peritoneal lavage CEA levels were correlated with survival in responders to conversion therapy (intraperitoneal and intravenous paclitaxel plus oral S-1) who subsequently underwent gastrectomy [135]. Recently, SYT13 mRNA in peritoneal lavages was also found to be associated with peritoneal recurrence [110]. Ber-EP4 has been an established marker for free peritoneal tumor cells and its positivity strongly correlates with poor prognosis [116,117,118,119]. *CDO1* gene promoter DNA methylation, a DNA marker assessed using quantitative methylation specific PCR, could be used to detect minimal residual disease of the peritoneum in GC patients [120]. Telomerase activity in peritoneal lavage was correlated with the presence and proliferating activity of peritoneal metastasis [112,113,114]. Furthermore, metabolic parameters were examined as indicators of PD. For instance, levels of lysophosphatidic acid were elevated in GC malignant ascites and significantly reduced after intraperitoneal chemotherapy [125]. Dopa decarboxylase levels showed sensitivity of 87% and specificity of 98% for diagnosis of synchronous PD [122]. Research advances in GC-derived peritoneal exosomes have involved the examination of exosomal biomarkers in peritoneal fluid. Recently, reduced expression of exosomal miR-29s were found to be associated with synchronous PD and PD recurrence in GC with serosal involvement [127].

### 4.3. Molecular Markers in Primary Gastric Cancer Tissues

Apart from biomarkers in peritoneal lavages, multiple molecular markers assessed using immunohistochemistry or RT-PCR associated with PD in primary tumor have been identified. Trypsinogen protein and trypsinogen-1 mRNA could be used for early diagnosis of PD in GC [109]. GC with high S100A4 expression showed association with PD and poor outcomes [129,130]. Through transcriptome analysis of 16 primary GC tissues, a group from Nagoya University identified and demonstrated SYT8 [132], troponin I2 [133] and SYT13 [111] as prognostic markers specific to peritoneal metastasis. Moreover, high mesothelin expression was found to be independently associated with poor relapse-free survival, overall survival and peritoneal recurrence in curatively resected stage III GC [131]. In another study, CEA and CK20 mRNA of gastric serosa from the primary GC were applied as biomarkers in a serosal stamp cytology to identify patients at high risk of peritoneal recurrence [108]. High telomerase activity in GC tissues tended to be associated with PD [115]. Bioinformatic analysis combined with cohort study data revealed that ARL4C expression was associated with PD risk and was a poor prognostic factor [128]. In a Chinese GC cohort of 74 patients, enrichment of *CDC27, MACF1* and *HMCN1* mutations was found in patients with peritoneal metastasis, while their prognostic significance remains unclear [136].

## 5. Conclusions and Future Perspective

PD is a major cause of mortality in GC patients, and it currently lacks effective treatment. Novel molecular classification schemes expanded our knowledge of the heterogeneity within GC. Specifically, gene expression-based ACRG classification explicitly differentiated a molecular subtype associated with peritoneal recurrence tendency. Recent reports of molecular characteristics, in particular, proteomics of DGC, have unveiled the heterogeneity within this histological type as being at significantly higher risk of PD. However, none of these studies assessed the PD risk or metastasis pattern that corresponds to the newly-proposed disease subtypes. Future studies should consider examining the specific PD-associated proteomic features in DGC or unspecified GC.

So far, molecular classifications using TCGA and ACRG have been the most important and recognized ones in GC, while other classifications have also shown correlation with clinical outcomes. In particular, MSS/EMT of the ACRG classification has been clearly associated with PD propensity. Meanwhile, multiple GC subtypes of other classification systems largely overlap with MSS/EMT, suggesting a trend towards higher PD risk. Based on these findings, targeted tests for *CDH1* mutations, E-cadherin aberrations, *CLDN18-ARHGAP* fusion, EMT-related gene expression or NGS, if possible, in addition to routine examinations, could help identify patients at high risk of PD. Development of a cost-effective panel representative of a PD-associated signature might allow a broader application in the clinical scenario. Furthermore, preoperative assessments of a biopsy specimen, circulating tumor cells or circulating tumor DNA are considerable measures to stratify PD risk in advance, informing treatment decisions regarding intraperitoneal therapy. Prospective clinical trials are required to validate the feasibility and instructive role of PD risk stratification.

Multiple PD-specific biomarkers identified from serum, peritoneal lavages and primary GCs could serve as candidate indicators for patient outcome and potential therapeutic targets for peritoneal carcinomatosis. However, except for serum tumor markers, most biomarkers are evaluated in retrospective cohort studies and have not been applied in routine tests. Further cohort validation and prospective large-scale studies are required for their translation to clinics. In addition, diagnostic or predictive biomarkers of PD might help guide personalized treatment. Intraperitoneal immunotherapy such as intraperitoneal infusion of chimeric antigen receptor (CAR)-T cells has shown promising results in preclinical studies [137,138]. There are ongoing phase 1 and 2 clinical trials aiming to evaluate the safety and efficacy of CAR-T cells targeting EpCAM (NCT03563326), CEA (NCT02349724) or mesothelin (NCT03941626) in treating advanced GC.

In short, risk prediction of PD based on molecular profiling and specific biomarker analysis is crucial to improved management of GC. Findings from clinical and multi-omic analysis suggest a subset of GC patients are at high risk of PD. An evidence-based approach to precise risk stratification is required. Multiple biomarkers have shown potential for PD prediction in GC, though most of them require further validation by clinical trials. Moreover, investigations into metastatic heterogeneity and intraperitoneal TME are relevant to improved understanding and management of peritoneal metastasis of GC.

## Figures and Tables

**Table 1:** Diagnostic and/or predictive biomarkers for peritoneal dissemination of gastric cancer.

Biomarker	Prognostic (Survival)	Diagnostic	Sample Type	Possible Assay Type for Biomarker Assessment	Reference
CEA	Positive	Positive	Serum, peritoneal lavage	IR, RT-PCR	[88,89,90,91,92,93,94,95,96,97,98]
CA12-5	Positive	Positive	Serum, peritoneal lavage	IR	[90,99,100,101]
CA19-9	Positive	Positive	Serum	IR	[88,89,99,102]
CA72-4	Positive	Positive	Serum	IR	[88,99,102]
CCL5	Positive	Positive	Serum, GC	IR, IHC	[103]
CK20, CEA	Positive	Positive	GC, Peritoneal lavage	RT-PCR	[104,105,106,107,108]
Trypsinogen-1	TBD	Positive	GC, peritoneal lavage	IHC, RT-PCR	[109]
SYT13	Positive	Positive	GC, peritoneal lavage	RT-PCR	[110,111]
Telomerase activity	TBD	Positive	GC, Peritoneal lavage	TRAP assay	[112,113,114,115]
Ber-EP4	Positive	Positive	Peritoneal lavage	ICC	[116,117,118,119]
CDO1 gene promoter DNA methylation	Positive	Positive	Peritoneal lavage	quantitative methylation specific PCR	[120]
IL-17	Positive	Positive	Peritoneal lavage	RT-PCR	[121]
Dopa decarboxylase	TBD	Positive	Peritoneal lavage	RT-PCR	[122]
MMP-7	Positive	Positive	Peritoneal lavage	RT-PCR	[123,124]
Lysophosphatidic acid	Positive	Positive	Peritoneal lavage	RT-PCR	[125]
CEA, CK20, survivin and MUC2	TBD	Positive	Peritoneal lavage	RT-PCR	[91]
CK20, FABP1, MUC2, TFF1 and MASPIN	Positive	TBD	Peritoneal lavage	ICC	[126]
Exosomal miR-29s	Positive	TBD	Peritoneal lavage	RT-PCR	[127]
ARL4C	Positive	Positive	GC	RT-PCR, IHC	[128]
S100A4	Positive	Positive	GC	RT-PCR, IHC	[129,130]
Mesothelin	Positive	TBD	GC	IHC	[131]
SYT8	Positive (PM-free survival)	Positive	GC	RT-PCR, IHC	[132]
Troponin I2	Positive (PM-free survival)	Positive	GC	RT-PCR	[133]

GC, gastric cancer; ICC, immunocytochemistry; IHC, immunohistochemistry; IR, immunoradiometric assay; PM, peritoneal metastasis; RT-PCR, reverse transcription polymerase chain reaction; TRAP, telomeric repeat amplification protocol; TBD, to be determined.
